# Tau Pathology in Chronic Traumatic Encephalopathy and Alzheimer's Disease: Similarities and Differences

**DOI:** 10.3389/fneur.2019.00980

**Published:** 2019-09-10

**Authors:** Atsuko Katsumoto, Hideyuki Takeuchi, Fumiaki Tanaka

**Affiliations:** Department of Neurology and Stroke Medicine, Graduate School of Medicine, Yokohama City University, Yokohama, Japan

**Keywords:** tau, traumatic brain injury, chronic traumatic encephalopathy, *cis* p-tau, prion

## Abstract

Traumatic brain injury (TBI) has been associated with the development of Alzheimer's disease (AD) because these conditions share common pathological hallmarks: amyloid-β and hyperphosphorylated tau accumulation. However, given recent data it is uncertain if a history of TBI leads to the development of AD. Moreover, chronic traumatic encephalopathy (CTE), caused by repetitive mild TBI and characterized by progressive neurodegeneration with hyperphosphorylated tau, has come to be recognized as distinct from AD. Therefore, it is important to elucidate the clinical outcomes and molecular mechanisms underlying tau pathology following TBI. We summarize the histopathological features and clinical course of TBI in CTE, comparing the tau pathology with that in AD. Following brain injury, diffuse axonal injury, and hyperphosphorylated tau aggregates are observed within a shorter period than in AD. Hyperphosphorylated tau deposition usually begins in the perivascular area of the sulci in the cerebral cortex, then spreads unevenly in the cortex in CTE, while AD shows diffuse distribution of hyperphosphorylated tau in the cortical areas. We also highlight the molecular profile of tau and the implications of tau progression throughout the brain in both diseases. Tau contains phosphorylation sites common to both conditions. In particular, phosphorylation at Thr^231^ triggers a conformational change to the toxic *cis* form of tau, which is suggested to drive neurodegeneration. Although the mechanism of rapid tau accumulation remains unknown, the structural diversity of tau might result in these different outcomes. Finally, future perspectives on CTE in terms of tau reduction are discussed.

## Introduction

Traumatic brain injury (TBI) is defined as damage to the brain caused by an impact such as a blow or jolt to the head. In contrast to the previous view that most people fully recover from mild TBI, a recent scoping review revealed that approximately half of patients experience serious long-term cognitive impairment, including problems with executive function, learning memory, attention, processing speed, and language function (1). A large cohort study using data from 2.8 million medical records showed that a single mild TBI was associated with a 20% greater risk of dementia (2). Moreover, emerging data have indicated that moderate and severe TBIs demonstrate a dose-response trend as risk factors for neurodegenerative diseases, including cerebral atrophy (3, 4), Alzheimer's disease (AD) (5), chronic traumatic encephalopathy (CTE) (6–10), and Parkinson's disease (PD) (11–13). However, the underlying mechanisms between TBI and these neurodegenerative diseases remain unknown. While tauopathy is a common pathological finding and there seems to be a close association between tau pathology following TBI and dementia, it has long been debated whether TBI can specifically lead to AD, or whether CTE following TBI can cause AD. Here we review both the pathological and molecular features of tau in TBI, including prospective therapeutic strategies.

## Tau Pathology

**Figure 1:**
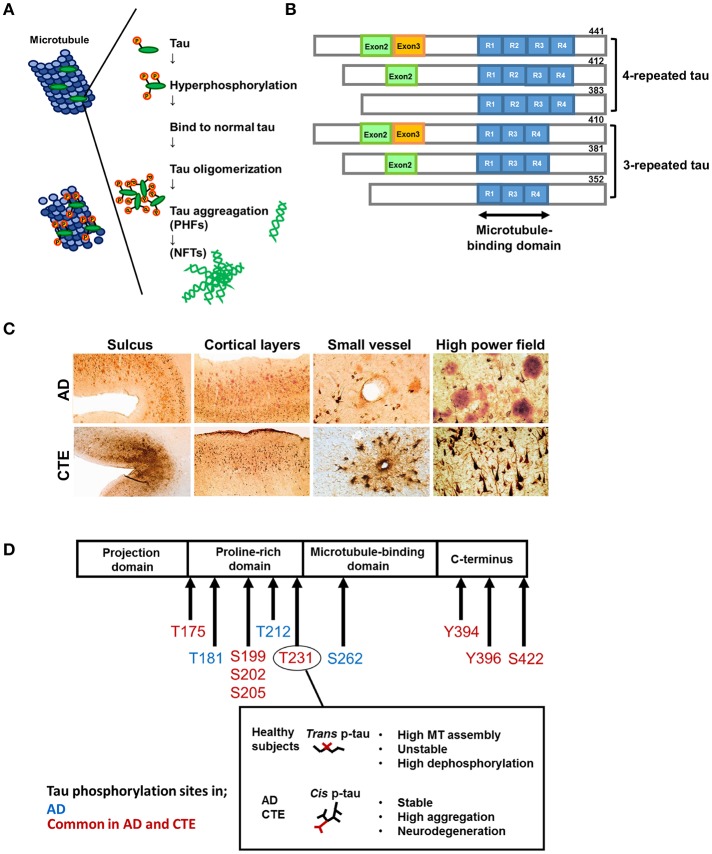
Formation of hyperphosphorylated tau aggregates. Under normal condition, the microtubule-associated protein tau is soluble and abundant in axons of neurons. In pathological conditions, tau can be hyperphosphorylated and dissociates from microtubules. Then hyperphosphorylated tau translocates to the cell body and aggregates into intracellular inclusions termed paired helical filaments (PHFs) and neurofibrillary tangles (NFTs). **(B)** Tau isoforms. There are six isoforms of tau in human brain. Tau isoforms with four microtubule binding domains, designated as 4R-tau, are accumulated in progressive supranuclear palsy (PSP) and corticobasal syndrome (CBS), whereas tau isoforms with three microtubule binding domains, designated as 3R-tau, are observed in Pick's disease. All six tau isoforms are involved in AD. This pattern is also detected in Down syndrome (DS), and amyotrophic lateral sclerosis and parkinsonism-dementia complex of Guam syndrome (ALS/PDC), and chronic traumatic encephalopathy (CTE) following TBI. **(C)** Characteristic pathology in AD and CTE. Upper panels: AD brains show diffuse cortical distribution of neurofibrillary tangles, preferentially distributed in laminae III and V without accentuation deep within sulci. Few fibrillary tangles exist around the small vessels. Double immunostaining demonstrates the coexsistence of abundant amyloid-β plaques (red) and interspersed PHF-1 neurofibrillary tangles (brown). Lower panels: In CTE, AT8 staining exhibits irregular cortical distribution of p-tau pathology with prominent subpial clusters of p-tau astrocytic tangles, focal accumulation deep within sulci, and neurofibrillary tangles in superficial cortical laminae II–III. Perivascular distribution of astrocytic tangles and neurofibrillary tangles are prominent in the small vessels. Double immunostaining reveals dense neurofibrillary tangles (brown) without amyloid-β plaques (red). Reproduced with permission from McKee et al. (8). **(D)** Representative tau phosphorylation sites in AD and CTE. CTE shares common phosphorylation sites with AD. Phosphorylation of tau at Thr^231^ enables the *cis*-*trans* conformational change of p-tau *Cis* and the *trans* formation of phosphorylated tau. *Trans* formation of p-tau, observed in healthy subjects, promotes microtubule assembly, and is critical for normal neuronal function. Phosphorylation status is unstable and easy to be dephosphorylated. *Cis* p-tau is stable and resistant to binding to microtubules, protein phosphatases, and degradation. In AD and TBI, *cis* p-tau is robustly accumulated and thereby causes and spreads tau aggregation, contributing to the development of neurodegeneration.

Tau is an abbreviated or alternative term for the microtubule-associated protein tau (MAPT). Microtubules are essential for the normal trafficking of cellular cargo in neuronal axonal projections (14). Under normal conditions, MAPT is a soluble protein that facilitates microtubule stabilization in cells, and is found in particularly high concentrations in neurons. In pathological conditions, tau can be more phosphorylated than normal (phosphorylation, Figure 1A). Hyperphosphorylated tau molecules dissociate from microtubules in the axon, translocate to the cell body and proximal dendrites, and aggregate into intracellular inclusions termed neurofibrillary tangles (NFTs), leading to impaired axonal function. Tau hyperphosphorylation itself decreases tau binding to microtubules and promotes tau fibrillization (15, 16). Furthermore, there is growing evidence that tau aggregates can recruit other tau aggregates to themselves and then spread to surrounding regions (17).

The term “tauopathy” was used for the first time in 1997 in the context of familial multiple system tauopathy with presenile dementia, a disease with abundant NFTs. Tauopathy is currently a collective designation of diseases, such as AD, Pick's disease, frontotemporal lobar degeneration (FTLD), progressive supranuclear palsy (PSP), and corticobasal degeneration, in which abnormal accumulation of phosphorylated tau (p-tau) protein in cell bodies is considered to be important in pathogenesis. Accumulated p-tau protein is observed as NFTs in neurons and glial cytoplasmic inclusions in astrocytes and oligodendrocytes. There are six isoforms of tau in the human brain (Figure 1B). Tau isoforms with three microtubule-binding domains, designated as 3-repeat tau (3R-tau), are generated by the splicing of exon 10 and are observed in Pick's disease (18), whereas tau isoforms with four microtubule-binding domains, designated as 4-repeat tau (4R-tau), accumulate in PSP and corticobasal degeneration (19). All 3R- and 4R-tau isoforms are contained in filamentous inclusions in AD, Down syndrome, CTE, and the amyotrophic lateral sclerosis and parkinsonism-dementia complex of Guam syndrome following TBI (details below) [(20–22); Table 1].

**Table 1:** Comparison of pathology between CTE and AD.

	**CTE**	**AD**
Regions of brain atrophy	Cavum septum pellucidum, frontal lobe, temporal lobe diencephalon, mammillary bodies, ventricular enlargement (23)	Diffuse (particularly medial temporal lobe) (21)
Tau protein	All six isoforms (3R- and 4R-tau) (24)	All six isoforms (3R- and 4R-tau) (24)
Tau progression	First perivascular area in the neocortex, locus coeruleus, diencephalon, and medial temporal lobe(particularly layers II and III) (6)	First in the brainstem, then the entorhinal cortex, amygdala, hippocampus, basal temporal cortex, insular cortex, and basal frontal cortex (particularly layersIII and V) (21)
p-tau deposition	Patchy irregular distribution in superficial layers of adjacent cortex (6)	Diffuse distribution in neocortex (21, 25)
Phosphorylation sites	T175, S199, S202, S205, T231, Y394, Y396, S422 (26–30)	T175, T181, S199, S202, S205, T212, T231, S262, Y394, Y396, S422 (31–34)
Axonal injury	Extensive in white matter tracts (6)	Sparse
Aβ	Relatively absent^36)^	Prominent (21)
TDP-43–positive inclusions	85% in the cortex, white matter, diencephalon, and brainstem (5–7)	15–75% in the hippocampus and frontal cortex (35, 36)

*Aβ, amyloid-beta; S, serine; T, threonine; TDP-43, TAR DNA-binding protein 43; Y, Tyrosine, 3R-tau, 3-repeat tau; 4R-tau, 4-repeat tau*.

## Pathological Comparison Between Tau in TBI and Alzheimer's Disease

### Single TBI

One of the reasons why TBI has been considered to increase the risk of AD is that they share similar pathological features and a clinical course involving a dementing process. Two main pathological hallmarks of AD are the extracellular deposition of amyloid-β (Aβ) aggregates and hyperphosphorylated tau (37). Diffuse axonal injury (DAI) and Aβ deposition were identified in up to 30% of patients who died acutely following a single TBI (38–41); these findings were present even just a few hours after the TBI occurred (39). By contrast, several studies with a short observation period failed to identify tau pathology (40, 42). More recent studies, however, showed that up to a third of patients with even a single episode of TBI developed NFT pathologies, and there seemed to be an association between even a single TBI and the development of dementia (43, 44). Similarly, severe TBI in wild-type mice induced progressive tau pathology that spread to the contralateral side of the injury (43). A recent proteomics study comparing diffuse and focal TBI patients showed heterogeneity among the different subtypes of TBI (45). In that study, the presence of DAI caused larger global alterations in the cortical tissue than focal TBI, resulting in increased production of proteins related to neurodegeneration and reduced protein production related to antioxidant defense (45). These findings suggest that even a single TBI can induce progressive tau pathology for years after the initial injury, especially in the presence of DAI.

### Repetitive TBI

Hyperphosphorylated tau aggregates and NFTs are often detected after TBI in combat-experienced military veterans and professional sports players at high risk of repetitive head injury, such as American football players, boxers, and wrestlers (6–10). These patients present clinically with mood and behavioral disorders and cognitive impairment known as CTE, previously termed “punch-drunk syndrome” or “*dementia pugilistica*” (7, 23, 46). Comprehensive analysis of post-mortem brains from individuals who experienced repetitive mild TBI revealed a strikingly high frequency of CTE (68/85 cases), with pathological changes distinct from those of other neurodegenerative diseases, including AD [Figure 1C; (8)]. As shown in Table 1, cavum septum pellucidum and enlargement of the lateral and third ventricles are often observed in CTE. Distinct diffuse cortical atrophy, which is characteristic of AD, is not common in CTE (47). Histologically, NFT is one of the most common pathological findings in CTE, and has been observed focally and perivascularly in the cerebral cortex, with a predilection for deep sulci in the superficial neocortical layers (layers II and III) [Figure 1C; (7–9, 24, 48)]. In the later stages of CTE, NFTs typically spread irregularly to the neocortex, medial temporal lobe, diencephalon, basal ganglia, and brainstem. By contrast, NFTs in AD first develop in the brainstem and entorhinal cortex (Braak stage I–II), then in the medial temporal lobe (Braak stage III–IV), and finally in the neocortex (Braak stage V–VI), where NFTs are evenly distributed in layers III and V (8, 25, 37, 49, 50). A previous study using 2-(1-{6-[(2-[F-18]fluoroethyl)(methyl)amino]-2-naphthyl}ethylidene) malononitrile ([F-18]FDDNP)-PET imaging, which identifies NFTs in living humans, demonstrated that professional American football players with suspected CTE had fibrillar insoluble protein aggregates in the brainstem white matter tracts, with axonal damage along subcortical and cortical brain areas. This radiological deposition pattern is consistent with paired helical filament (PHF)-tau distribution in the autopsy samples from mild TBI patients. Importantly, these pathological changes in TBI are not consistent with Braak stage in AD (51, 52). Given these findings, it is plausible that CTE may occur frequently in cases of repetitive TBI. Since CTE is characterized by the distinct distribution of hyperphosphorylated tau pathology seen in AD, we discuss the differences in tau levels in biofluids in these diseases, including the possibility of utilizing tau as a biomarker.

## Tau Protein as a Biomarker

The clinical features of CTE are wide-ranging, and its diagnosis is made based on pathological findings in brain biopsy samples and at post-mortem examination. Therefore, prognostic biomarkers for CTE and TBI-related AD have been required.

The serum levels of total tau (t-tau) in concussed sports players increased in the acute stage following TBI, and decreased in the subacute stage (53). Similarly, serum p-tau (T231 and S202) and t-tau were elevated in severe human TBI and in a rodent model of repetitive mild TBI during both the acute and subacute periods (54–56). Acute elevation of both p-tau levels and the p-tau/t-tau ratio was found in TBI patients with poor outcomes (55). In addition, the recovery of serum t-tau to pre-injury levels correlated well with good outcomes (57). Elevations of plasma t-tau, p-tau (T231), and the p-tau/t-tau ratio were also observed in the chronic phase (6–18 months) following moderate-to-severe TBI and in military personnel with repetitive TBI (58, 59). While these data can help predict the short-term outcomes of TBI, they cannot clarify the long-term prognosis or identify CTE.

Stern et al. found that higher plasma exosomal tau levels in professional football players were associated with poor memory and psychomotor speed (60). Similar findings were seen in military personnel (61). Since exosomes are very stable, cross the blood–brain barrier, and reflect their cellular origin, exosomal tau may serve as an ideal predictor of CTE. The ultrasensitive tau seed amplification assay detected that seeding activity of tau aggregates in AD and CTE was markedly higher than in other tauopathies, such as Pick's disease, and seed concentrations in the brains of two CTE cases were comparable to the lowest concentrations in AD brains (62). These methods might be useful to distinguish CTE from AD.

## Conformational Variations of Hyperphosphorylated Tau

In most TBI cases, tau phosphorylation in the brain occurs as early as a few hours after the injury and then spreads at a high density within a shorter period than in AD (44). The mechanism underlying the rapid protein accumulation following TBI remains unknown. The molecular profile and function of tau might be different between AD and other tau-related disorders, including TBI. Analysis of tau phospho-epitopes in AD, FTLD, PSP, and CTE has shown that each neurodegenerative disease has a specific profile of p-tau formation (63–66). In addition, the misfolding and hyperphosphorylation statuses of tau proteins depend on mutations or different isoforms of tau, which may result in phenotypic differences between AD and other tau-related disorders (67). For example, mutated FTLD-tau proteins change their conformation without hyperphosphorylation (16, 26), which has not been observed in AD (67, 68). Tau phosphorylation sites in AD have been identified on serine (Ser) or threonine (Thr) residues that precede a Pro residue (31, 32). Three sites of tau phosphorylation, namely, Thr^231^, Thr^181^, and Ser^199^, serve as biomarkers for AD (27, 33, 34). In particular, hyperphosphorylation of tau at Ser^199^/Ser^202^/Thr^205^ or Thr^212^/Thr^231^/Ser^262^ is sufficient to induce microtubule instability that results in cell death (33, 34). Additionally, tau phosphorylated at Tyr^394^ has also been reported in PHFs from AD brains (28). In CTE, phosphorylation at Thr^175^ and Ser^422^ has been reported in addition to that at Ser^199^and Thr^231^ (29–31). A study in mice reported that TBI triggered calpain-2 activation and resulted in increased tyrosine phosphorylation of kinase c-Abl at Tyr245, which enhanced its kinase activity and p-tau at Tyr^394^ (69). Other common phosphorylation sites (Ser^199^, Ser^202^/Thr^205^, and Ser^396^) were also found in both AD and CTE (70). Whole RNA sequencing analysis of post-mortem brain tissue revealed that the expression of *PPP3CA*, which encodes a calcium-dependent, calmodulin-stimulated protein phosphatase, was decreased in CTE compared to normal controls, and the PPP3CA protein level was inversely correlated to the p-tau (Ser^202^/Thr^205^) level (70).

The same tau epitopes were found to map to filamentous tau inclusions in CTE and AD brains, although the abnormal tau proteins from CTE brains did not overlap with the six abnormally phosphorylated tau isoforms in AD (25). Similarly, insoluble tau protein from CTE brains contained all six isoforms, while an AD case contained the three isoforms that comprise PHFs (31). A recent study showed that phosphorylation of tau at Thr^231^ enabled the *cis*-*trans* conformational change of p-tau [Figure 1D; (71)]. The *cis* conformation of p-tau appears in neurons within hours after TBI, prior to the formation of tau oligomers, pre-fibrillary tangles, and NFTs, and results in axonal disruption (71). In both a TBI mouse model and TBI patients, *cis* p-tau not only triggered neurotoxic effects, but also spread to other brain regions, including the hemisphere contralateral to the injury, causing cognitive impairment (71). These data suggest that *cis* p-tau functions as a driver of neurodegeneration. Indeed, p-tau with the *cis* conformation, but not the healthy, physiological *trans* form, is detectable in both CTE and AD patients (72). Since the molecular weight of insoluble p-tau (Ser^199^) differed between CTE and epileptic brains (29), tau conformation may differ depending on disease type. A more precise understanding of the structural diversity of tau might clarify the basis for the heterogeneity of tau-related pathologies and the molecular differences between CTE and AD.

## Prion-Like Propagation of Tau Pathology in TBI

In prion disease, misfolded prion proteins act as seeds to initiate the misfolding and aggregation of the native prion protein (73). Eventually, the long polymers undergo fragmentation to release more seeds, which accelerate the rate of prion propagation (73). Tau pathology (especially 4R-tau) in AD appears to develop hierarchically along anatomical connections, which suggests cell-to-cell transfer of toxic tau through neuronal cell contacts (74, 75). Studies of animal models imply that tau transfer may be partly mediated by a prion-like templated misfolding of tau (65, 76–80). Indeed, inoculated misfolded cellular prion protein (PrP^c^) can promote Aβ aggregation in AD mice by cross-seeding (81), accelerating tau hyperphosphorylation (82, 83). As such, increased levels of total and phosphorylated tau following severe TBI were associated with PrP^c^ production levels (58). Inoculation of brain homogenates from a mouse with severe TBI into the hippocampus and cerebral cortex of wild-type mice enhanced tau propagation and led to memory deficits; similar findings in the same study were observed using brain homogenates obtained contralateral to the side of the TBI, which supports a prion-like propagation mechanism of tau (43). Since misfolded tau aggregates in AD and CTE were replicated in cells expressing both 3R- and 4R-tau isoforms, but not cells expressing either 3R-tau or 4R-tau, the propagation properties of misfolded tau aggregates in AD and CTE are distinct from those in Pick's disease and PSP (22).

It is also plausible that hyperphosphorylated tau deposition promotes the accumulation of other aggregate-prone proteins. In addition to tauopathy, CTE is accompanied by other proteinopathies such as amyloidopathy and TAR DNA-binding protein 43 (TDP-43) proteinopathy (7–9), the latter of which was originally thought to be a specific marker for ALS that forms ubiquitinated inclusions (35, 36). TDP-43 inclusions were found in 85% of CTE cases [(8, 84, 85); Table 1]. Especially in the late stage of CTE, TDP-43 inclusions are severe in the cortex, white matter, diencephalon and brainstem, a distribution that is similar to that of NFTs. Partial co-localization of TDP-43 with tau was detected in the late stage of CTE (8). Like in AD, aggregated tau propagation in TBI might promote TDP-43 accumulation. However, it is not yet fully clear why the pathological tau distribution differs between these diseases.

## Does TBI Accelerate the Development of Alzheimer's Disease?

A retrospective analysis of TBI patients demonstrated that the onset of AD was significantly earlier in those who survived from TBI (86, 87), especially in men (88). Examination of autopsy samples in a small cohort showed an AD prevalence of 22% in TBI patients, which was significantly higher than the 14% observed in the general population over age 70 (89). Since the concept of mild cognitive impairment (MCI) was accepted (90), it has been recognized that there is a link between TBI and the early diagnosis of MCI, although no significant association has been observed between TBI and the rapid progression from MCI to AD (91). Similarly, retired American football players with a history of at least three concussions demonstrated a 5-fold greater prevalence of MCI (92). Both moderate and severe TBI in military veterans were also associated with an increased risk of developing AD, with onset accelerated by 2 years (93–95). Accordingly, TBI is associated with increased risks of MCI and AD, which also accelerate AD development. Interestingly, Kanaan et al. have documented the mixed phenotype of AD and CTE (32), where both AD and CTE may be identified in the same patient. However, it remains unclear whether AD develops concurrently with CTE or if instead CTE induces AD.

## Future TBI Treatment Prospects Considering TAU Pathology

There is a growing interest in pathogenic tau as a therapeutic target, since ablation or reduction of tau restores cognitive impairment following TBI (96, 97). Different tau kinases have been shown to control the binding ability of tau to microtubules (98). For example, when tau is first phosphorylated by glycogen synthase-3 or cyclin-dependent kinase-5, its binding ability to microtubules is inhibited by 79% (98). In addition, the combination of these kinases accelerates phosphorylation at Thr^231^ and Ser^262^. Replacement of these phosphorylation sites or inhibition of phosphorylation kinases has been attempted *in vitro* and in AD murine models (34, 99, 100). In TBI, the finding that calpain-2 activation by TBI accelerates tau phosphorylation at the Tyr^394^ site (28) suggests that inhibiting calpain-2 or its pathway may be a promising treatment strategy.

Given that *cis* p-tau is a precursor of tau pathology, proline isomerase Pin1, which converts p-tau from pathogenic *cis* to physiologic *trans*, may be a viable drug target (71, 101, 102). Remarkably, a neutralizing antibody for *cis* p-tau that acts both extracellularly and intracellularly facilitated tau binding to microtubules, restored normal tau function, and prevented tau spread, axonal damage, and neuronal degradation in mouse models of both AD and CTE (71, 102, 103). The *cis* to *trans* transition mediated by Pin1 also enhanced the degradation of p-tau (104, 105). In addition, therapeutic administration of the *cis* p-tau neutralizing antibody after TBI suppressed *cis* p-tau induction in a TBI mouse model (72). Further development of therapeutics for clinical application are expected.

## Conclusion

We reviewed the distribution, molecular characteristics, and development of tau pathology in TBI, with a particular emphasis on CTE. Although the observed tau isoforms are common between AD and CTE, the brain lesions involving tau are distinct in both diseases, and each tau progression pattern is different. Conformational variance may influence the rapid tau development following TBI. Epidemiological studies have shown that TBI may be a risk factor for AD as well as CTE, and more interestingly, the mixed pathology of AD and CTE coexists in some TBI patients. However, the reasons why clinical and pathological outcomes differ between patients have yet to be fully elucidated. Further investigation is needed to provide an effective treatment for TBI.

## Author Contributions

AK wrote the manuscript. HT and FT edited the manuscript.

### Conflict of Interest Statement

The authors declare that the research was conducted in the absence of any commercial or financial relationships that could be construed as a potential conflict of interest.

## Abbreviations

AD: Alzheimer's disease
CTE: chronic traumatic encephalopathy
DAI: diffuse axonal injury
FTLD: frontotemporal lobar degeneration
MCI: mild cognitive impairment
p-tau: phosphorylated tau
TBI: traumatic brain injury
TDP-43: TAR DNA-binding protein 43.
